# Supplementary material for: RAD51C-XRCC3 structure and cancer patient mutations define DNA replication roles
Source: Nat Commun. 2023 Jul 24;14:4445. doi: 10.1038/s41467-023-40096-1 (PMC10366140; doi:10.1038/s41467-023-40096-1)
Supplement: Supplementary file 1 — Supplementary Information [file 41467_2023_40096_MOESM1_ESM.pdf]

Supplementary Information

Supplementary Figures

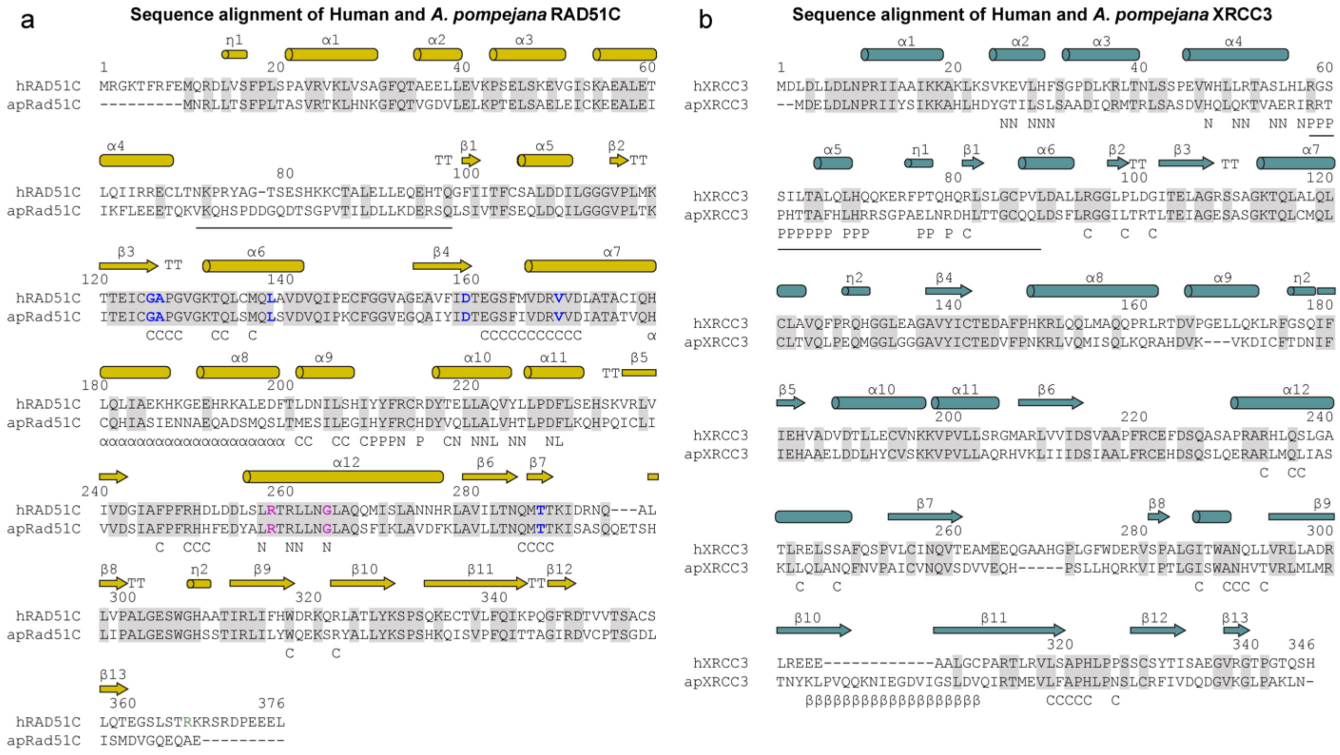

Supplementary Fig. 1 RAD51C and XRCC3 sequence alignments.
Protein sequence alignments of human (numbered) and *A. pompejana* **a**, RAD51C and **b**, XRCC3. Secondary structural elements seen in the structures are indicated above the sequence, and residues involved in CX3 interfaces are indicated below with N, for the XRCC3 NTD, P, for the XRCC3 polymerization motif, and C for the CX3 CTD interface, respectively. Black lines underneath highlight the linker containing the PM that separates the NTD from CTD. The RAD51C cancer variants are highlighted as colored throughout figures.

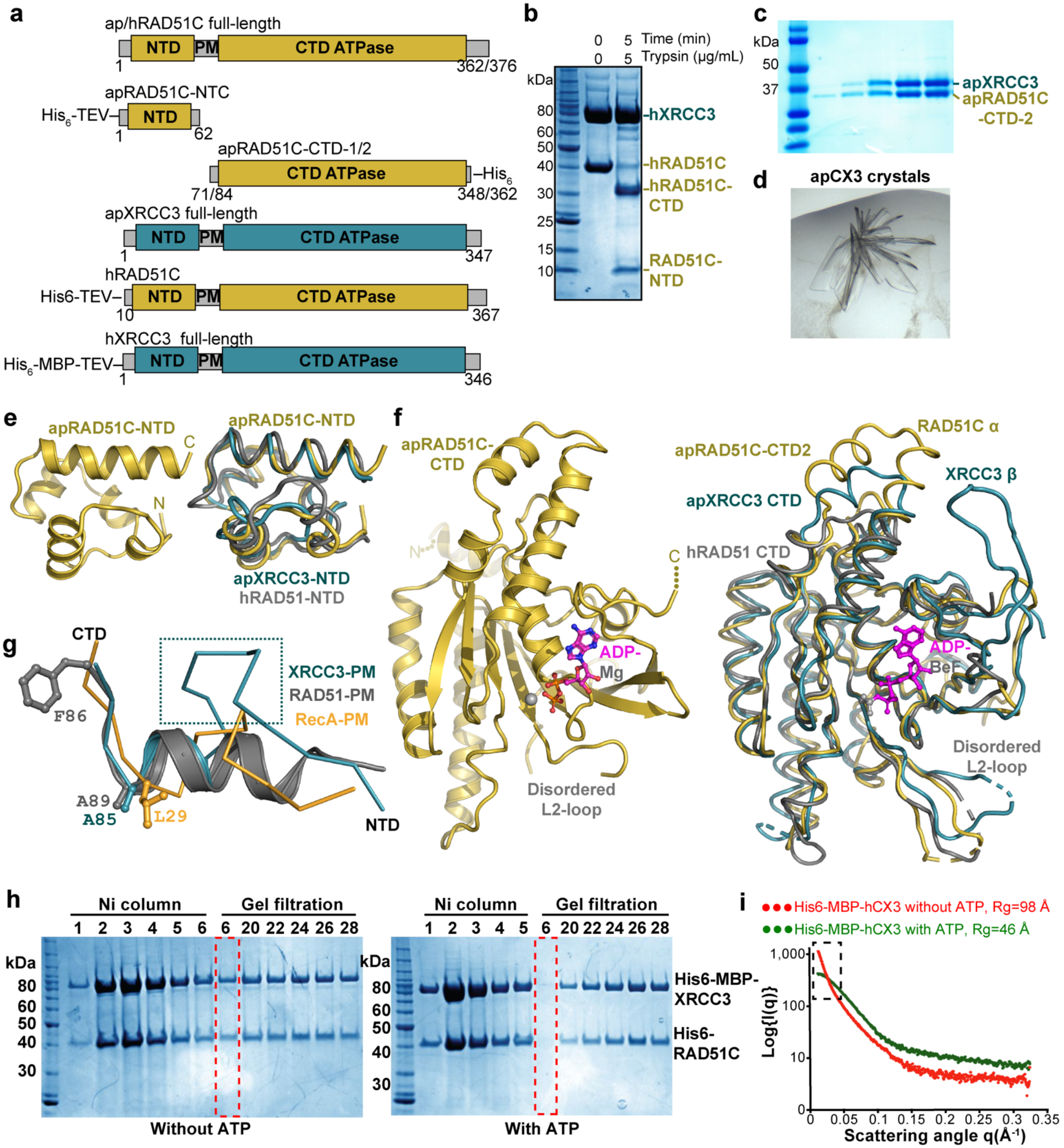

Supplementary Fig. 2. RAD51C and XRCC3 constructs, protein purification, limited proteolysis, RAD51C NTD and CTD structures.

a, Schematics of the constructs used for structural and biochemical work, with the RAD51C and XRCC3 amino acids and purification tags encoded in each construct highlighted. apRAD51C-CTD-1 was used to determine the structure of the RAD51C CTD, and RAD51C-CTD-2 was co-expressed with full length XRCC3 to make the apCX3 core.

b, Limited proteolysis of 1 mg/mL of hCX3 complex, prior to purification tag removal using the indicated concentration of trypsin and time at 4℃, was visualized by SDS-PAGE and coomassie blue staining. Representative image of at least 3 independent experiments.

c, SDS-PAGE and coomassie blue staining analysis of purified stoichiometric apCX3 core complex. Representative image of at least 3 independent experiments.

d, Representative image of apCX3 crystals.

e, Cartoon representation of the apRAD51C NTD crystal structure (top), and superposition of the NTDs of apRAD51C, apXRCC3 and hRAD51 (bottom).

f, Cartoon representation of the apRAD51C CTD crystal structure (left), and superposition of the CTDs of apRAD51C (from the CX3 complex), apXRCC3 and hRAD51 (right). The DNA binding L2-loop and unique RAD51C and XRCC3 structural elements are labeled.

g, Superposition of XRCC3, RAD51 and RecA polymerization motifs (PM), with key interface residues highlighted. A six amino acid insertion in the XRCC3 PM compared to RAD51 and RecA is highlighted by a dashed box.

h, Coomassie stained SDS-PAGE analysis of indicated Ni-column and gel filtration fractions of His_6-MBP-tagged CX3 without and with ATP, gel filtration void volume fractions are indicated with the red dashed box. Representative images from at least 3 independent experiments.

i, SAXS analysis of His_6-MBP-tagged CX3 without and with ATP. The dashed box highlights the region of the scattering curve that is most influenced by aggregation.

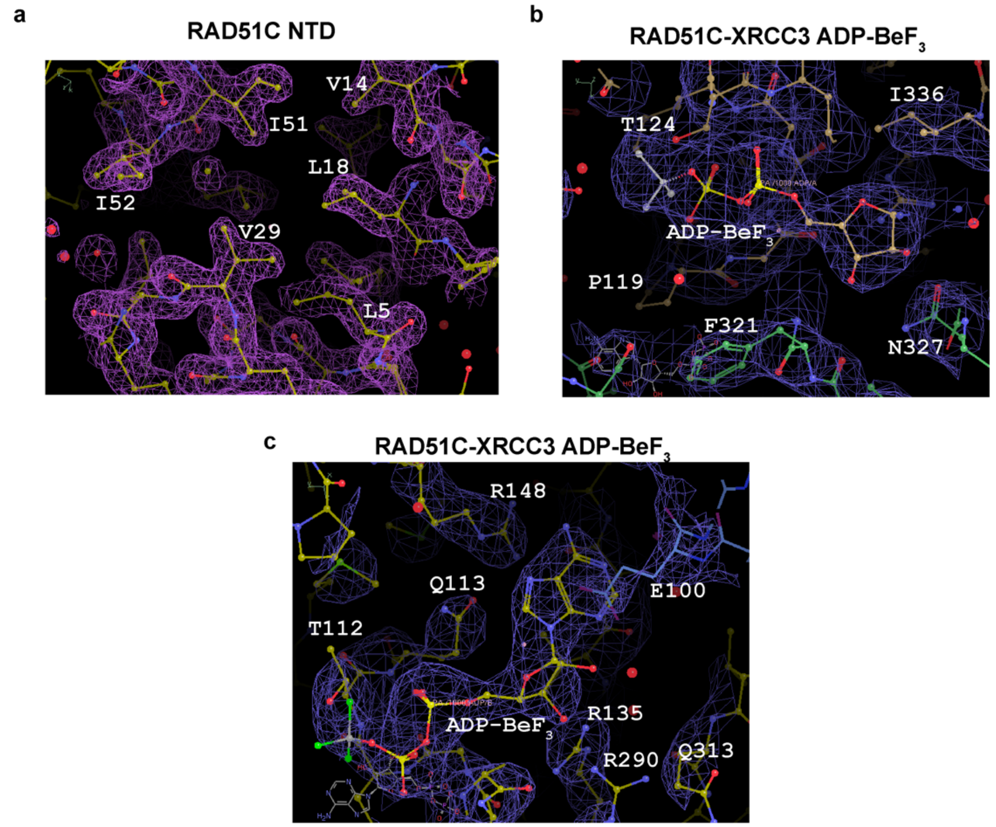

Supplementary Fig. 3. Representative electron density for apRAD51C NTD and apCX3 X-ray crystal structures.

All electron density maps are 2FoFc composite omit maps generated in Phenix and contoured at 1.5σ.
a, apRAD51C NTD electron density (purple mesh) with modelled residues coloured in yellow (carbon), oxygen (red) and nitrogen (blue). Clearly visible residues are labeled to highlight what region of the protein is shown.
b, apCX3 electron density (blue mesh) for nucleotide bound at the RAD51C-XRCC3 CTD interface with modelled residues coloured in tan (carbon, RAD51C), green (XRCC3), oxygen (red) and nitrogen (blue). ADP-BeF_3 is coloured in tan (carbon), red (oxygen), blue (nitrogen), yellow (phosphate) and light blue (BeF_3). Clearly visible residues are labeled to highlight what region of the protein is shown.
c, apCX3 electron density (blue mesh) at the XRCC3 nucleotide binding interface with modelled residues coloured in yellow (carbon), oxygen (red) and nitrogen (blue). ADP-BeF_3 is coloured in yellow (carbon and phosphate), oxygen (red), nitrogen (blue) and light blue and green (BeF_3). Clearly visible residues are labeled to highlight what region of the protein is shown, and E100 in blue is from a symmetry-related molecule.

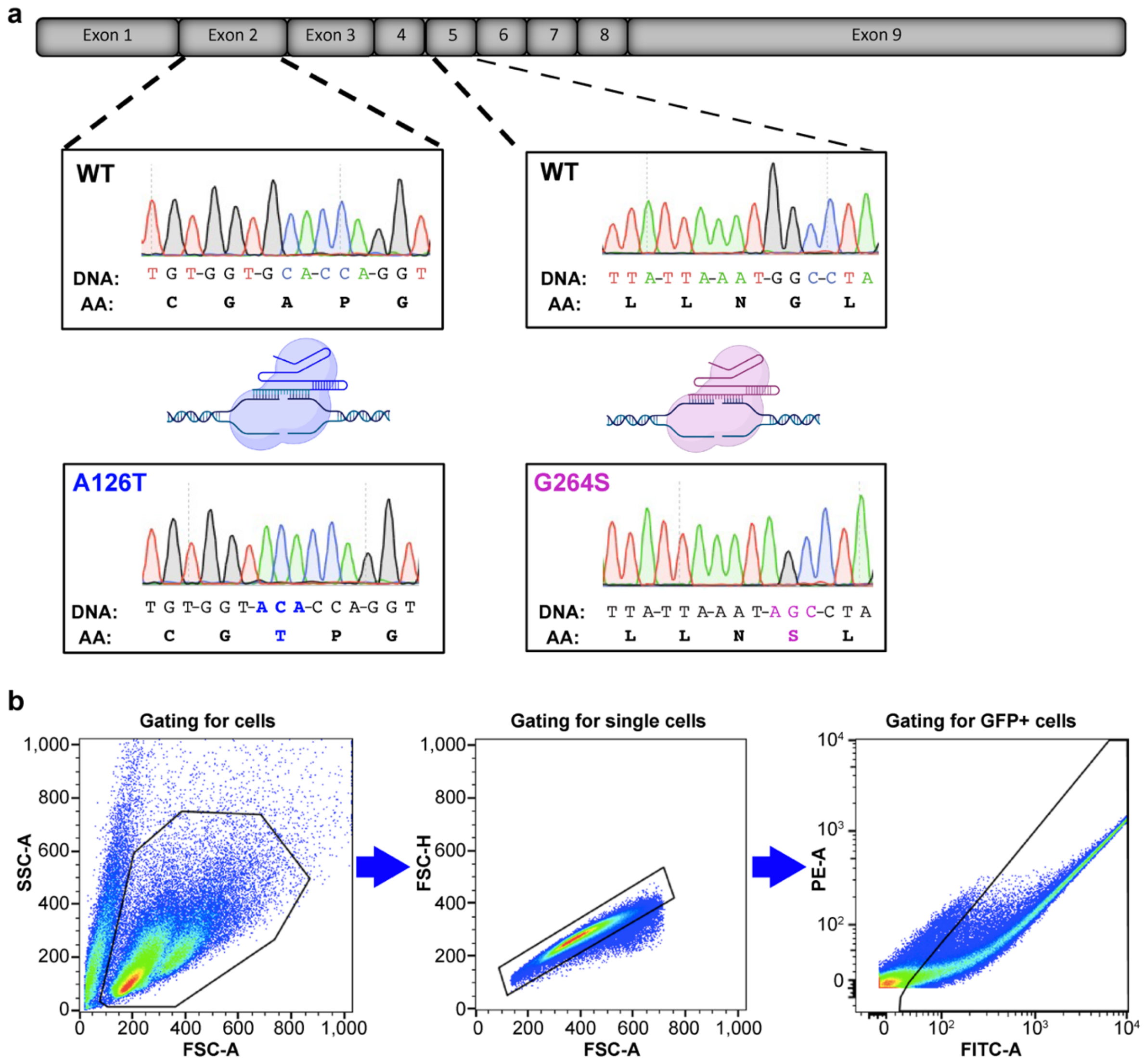

Supplementary Fig. 4 Isogenic HAP1 cells containing cancer variants.

a, Schematic representation of RAD51C cDNA (top). Experimentally determined Sanger sequencing peaks for wild-type RAD51C exons 2 and 5 (middle) and RAD51C variants A126T (bottom left) and G264S (bottom right) following CRISPR/Cas9 mediated gene editing of HAP1 cells. Gene editing schematics created with http://BioRender.com.

b, Gating strategy for Dr-GFP Assay, which are sequentially gated for live cells (left panel), followed by single cell (middle panel) and GFP positive cells (GFP+, right panel). FSC-A denotes forward scatter area, SSC-A side-scatter, FSC-H forward scatter height, PE-A Phycoerythrin channel area, FITC-A Fluorescein isothiocyanate channel area.

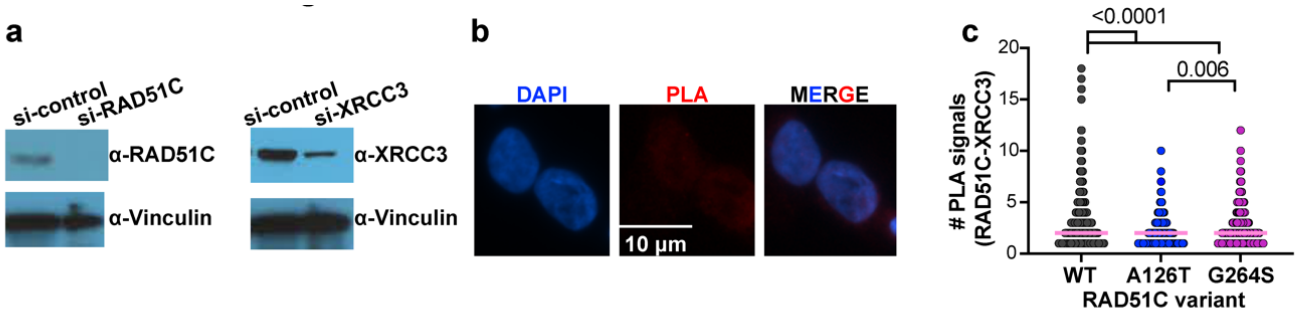

Supplementary Fig. 5 Controls for RAD51C-XRCC3 protein interaction studies.

a, Immunoblots of siRNA knockdown of RAD51C or XRCC3 proteins in HAP1 cells, verifying antibody target specificity. Vinculin is shown as loading control. Representative image of 2 biological repeats.

b, The absence of PLA signals in reactions with primary XRCC3 but leaving out primary RAD51C antibody further verifies the specificity of the PLA signals between XRCC3 and RAD51C. Representative image of 3 biological repeats.

c, Quantification of RAD51C-XRCC3 PLA signals in unchallenged human HAP1 cells containing indicated variant RAD51C. Pink bar denotes median. *P* values (<0.0001 between WT and other variant, 0.006 between A126T and G264S) were calculated using an unpaired, two-sided Student T-test, n(WT)=215, n(A126T)=90, n(G264S)=110, derived from 4 independent biological experiments.

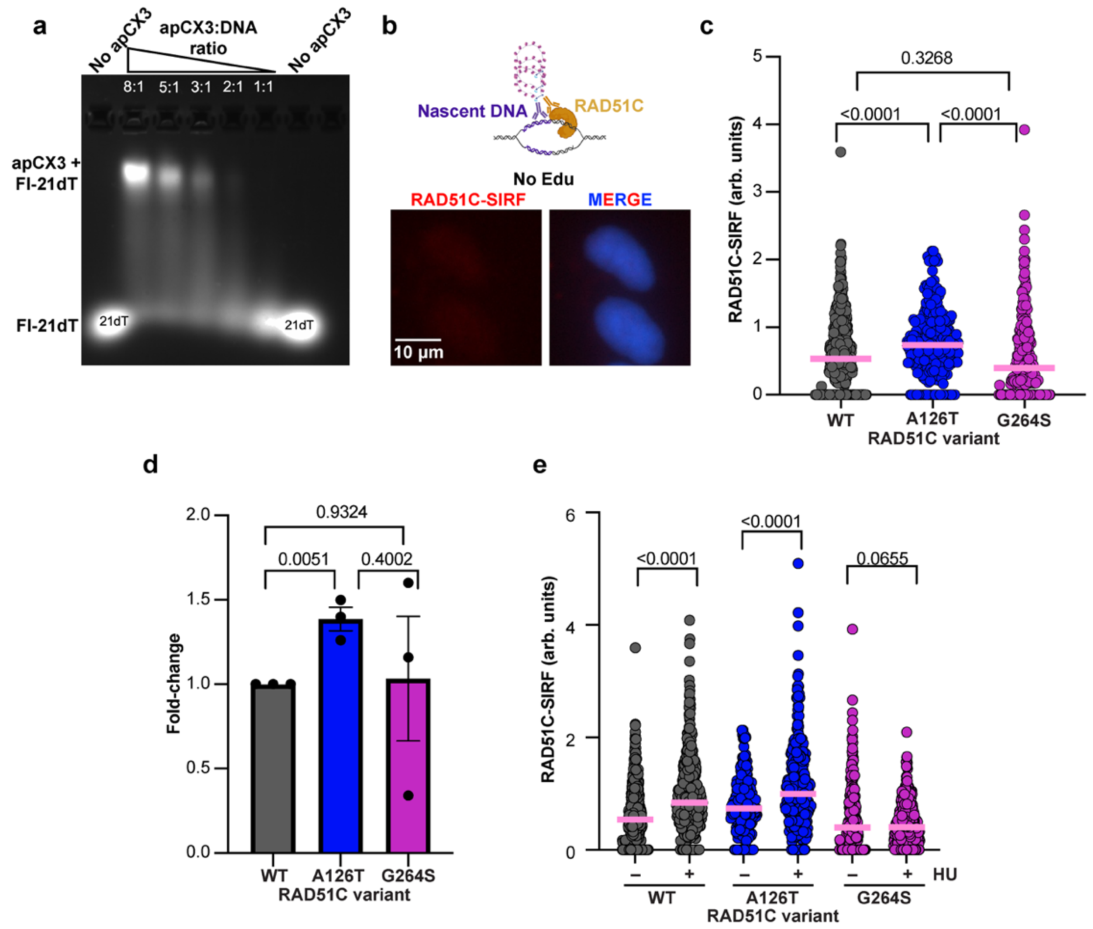

Supplementary Fig. 6 Controls for RAD51C-SIRF DNA interaction studies.

a, Electromobility shift assay for apCX3 ssDNA binding. Ratios of apCX3:DNA substrate concentrations are indicated and compared to reactions with no apCX3. Representative image of at least 2 independent experiments.

b, The absence of RAD51C-SIRF signals in reactions without EdU verifies the specificity of the RAD51C-SIRF signal. Representative image of 3 biological repeats. Top, graphical schematic of a RAD51C-SIRF reaction created with http://BioRender.com.

c, Quantification of RAD51C-SIRF signals in unchallenged human HAP1 cells containing indicated variant RAD51C shows similar results in wild-type and G264S cells, and slightly elevated associations for RAD51C A126T mutated cells. Pink bar denotes median. (arbitrary units, arb. units). *P* values (indicated above brackets in the figure panel) were calculated between each comparison using the unpaired, two-tailed Mann-Whitney test, n(WT)=568, n(A126T)=249, n(G264S)=241, derived from 3 independent biological experiments.

d, Fold-change of average RAD51C-SIRF signals in unchallenged HAP1 cells with indicated RAD51C variants. *P* values were calculated using an unpaired Student T-test, n=4 independent biological experiments. Data is presented as mean +/- SEM.

e, Replotting of RAD51C-SIRF data from Supplementary Fig. c and Fig. 4f with and without replication stalling (200 μM hydroxyurea) to visualize the relative increased in wild-type and RAD51C A126T binding to stalled forks, but not with RAD51C G264S. *P* values (shown above brackets on the figure panel) were calculated between each comparison using the unpaired, two-tailed Mann-Whitney test, n(WT)=568, n(A126T)=249, n(G264S)=241for nontreated and n(WT)=360, n(126T)=341, n(G264S)=415 for HU treated samples, derived from 3 independent biological experiments.

a

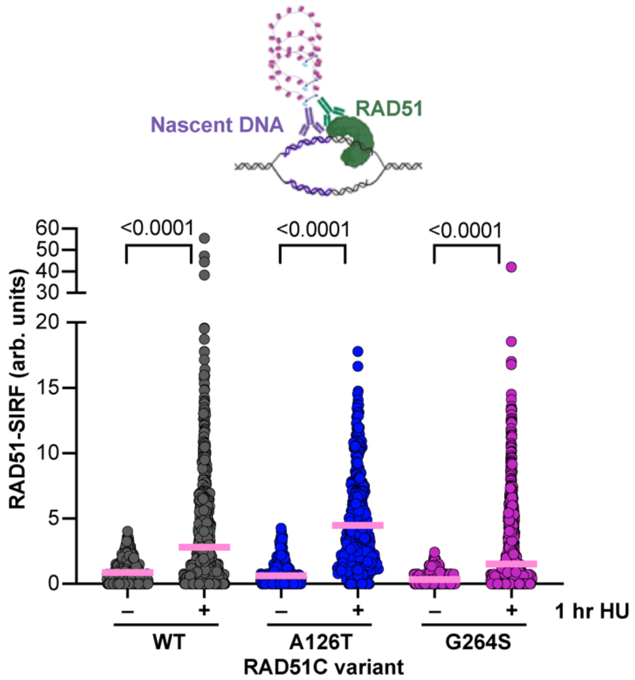

b

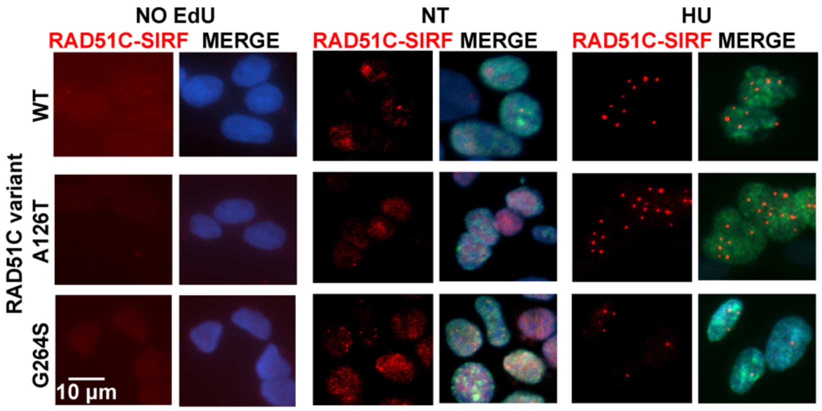

Supplementary Figure 7 Controls for RAD51-SIRF DNA interaction studies

a, Quantification of RAD51-SIRF signals in unchallenged human HAP1 cells containing indicated variant RAD51C and replotting of RAD51-SIRF data after 1 hour replication stalling from Fig. 5e,f shows a stark increase in RAD51-SIRF signals. Signals are normalized to available nascent EdU (arbitrary units, arb. units). *P* values (shown above brackets on the figure panel) were calculated between each comparison using unpaired, two-tailed Mann-Whitney test, n(WT)=422, n(A126T)=191, n(G264S)=297 for nontreated and n(WT)=342, n(A126T)=463, n(G264S)=701 for HU treated samples, derived from 3 independent biological experiments. Top, graphical schematic of a RAD51-SIRF reaction created with http://BioRender.com.

b, Representative images of RAD51-SIRF assays from 3 biological repeats with no EdU, showing specificity of the assay, and without or with 200 µM hydroxyurea in human HAP1 cells containing indicated variant RAD51C.

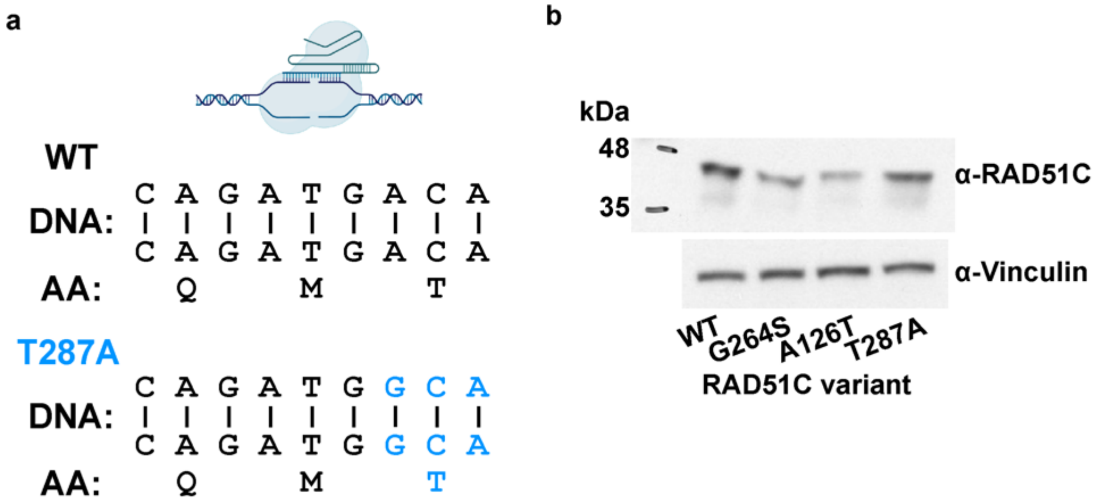

Supplementary Fig. 8 Isogenic HAP1 cells containing RAD51C T287A.

a, Nucleotide sequences for CRISPR/Cas9 edited RAD51C T287A HAP1 cells (bottom) showing the A to G nucleotide change compared to wild-type RAD51C (top) resulting in a threonine to alanine amino acid change at position 287. Top, gene editing schematic created with http://BioRender.com.

b, Western blot of RAD51C and Vinculin in wild-type and indicated RAD51C variant HAP1 cells. Representative image of 2 biological repeats.

.

Supplementary Table 1. X-ray crystallography data collection and refinement statistics of the apCX3, apRAD51C CTD and NTD structures determined in this study.

	apCX3 core	apRAD51C-CTD	apRAD51C-NTD
**Data collection**			
Space group	P 21 21 21	P 32 2 1	P 32 2 1
Cell dimensions			
a, *b*, *c* (Å)	65.94, 112.30, 259.50	96.03, 96.03, 55.68	45.61, 45.61, 111.41
α, β, γ (°)	90, 90, 90	90, 90., 120	90, 90, 120
Resolution (Å)	38.33 – 2.60 (2.67 – 2.60)	36.36 – 2.30 (2.38 –2.30)	32.21 – 1.60 (1.65 – 1.6)
R_{sym} or R_{merge}	0.214 (3.455)	0.022 (0.323)	0.085 (0.64)
$I / \sigma I$	15.5 (1.15)	15.31 (2.23)	6.00 (1.2)
Completeness (%)	99.9 (99.4)	99.69 (99.55)	95.64 (88.11)
Redundancy	26.44 (26.49)	2.0 (2.0)	14.2 (14.0)
**Refinement**			
Resolution (Å)	2.60	2.30	1.60
No. reflections	56007	13402	17625
R_{work} / R_{free}	0.187 / 0.232	0.188 / 0.237	0.156 / 0.196
No. atoms	14549	2097	1240
Protein	14192	2025	1035
Ligand/ion	182	28	1
Water	175	44	205
B-factors			
Protein	78.88	29.71	36.00
Ligand/ion	55.57	52.71	16.26
Water	60.35	53.28	48.03
R.M.S deviations			
Bond lengths (Å)	0.003	0.016	0.012
Bond angles (°)	0.854	1.87	1.10

*Values in parentheses are for highest-resolution shell. All data was collected from single crystals

Supplementary Table 2: SAXS reporting data

**Sample details**	**hCX3**
Organism	*Homo sapiens*
Source	Insect cell expressed then purified
Description: sequence & ligands	O43502-1 (RAD51C residues 10-367, His-tag removed). O43542-1 (XRCC3 residues 1-336, His-MBP tag. Nucleotide (ATP mimic) bound
Extinction coefficient ε [Abs 0.1% (w/v)]	0.521
Molecular mass (Da)	77,756
Concentration (range/values) measured	1, 2, 4 mg/mL (batch collection)
Solvent composition	10 mM HEPES pH8, 100 mM NaCl, 2.5 mM ATP, 2.5 mM $MgCl_2$, and 0.1 mM $NaVO_4$
**SAXS data collection parameters**	
Instrument/data processing	Sibyls beamline 12.3.1 at the Advanced Light Source (Berkeley, USA) with Dectris Pilatus3 2M Detector
Wavelength	1 Å
Exposure time, number of exposures	0.2 & 1 second exposures for each concentration
Sample configuration	Mica coated syringe
Sample temperature (ºC)	20
**Software employed for SAS data reduction, analysis and interpretation**	
SAXS data reduction	Radial averaging & subtraction done using established programs at Sibyls beamline 12.3.1
Basic analysis & merging of curves	Scatter & PRIMUS
Model building, molecular dynamics & minimal ensemble search	iTasser and BILBOMD
**Structural parameters**	
Guiner Rg (Å) / I(0)	36.21 +/– 0.38 / 58.53 +/– 0.48
P(r) Rg (Å) / I(0)	34.64 / 56.9
Dmax (Å)	110
Porod volume	135,065
Experimental molecular weight	78.5 kDa
**SASBDB accession code**	**SASDS36**

Supplementary Table 3: Details of antibodies used in this study

Antibody name	Clone	Company	Catalog no.	Dilution	Application
mouse anti-biotin	BN-34	Sigma-Aldrich	SAB4200680	1/100	SIRF
rabbit anti-biotin	D5A7	Cell Signaling Technology	5597	1/200	SIRF
rabbit anti-XRCC3	n/a	Abnova	PAB24835	1/100	PLA
mouse anti-RAD51C	3F3-5C6	Abnova	H00005889-M01	1/100	PLA/SIRF
rabbit anti-RAD51C	n/a	Abcam	ab72063	1/100	PLA/SIRF
mouse anti-RAD51	14B4	Abcam	Ab213	1/200	SIRF
mouse anti-RAD51C	2H11/6	Novus Biologicals	NB100-177	1/1,000	Immuno-blotting
rabbit anti-XRCC3	n/a	Novus Biologicals	NB100-165	1/1,000	Immuno-blotting
anti-IDU/BrdU	B44	Beckton Dickinson	347580	1/50	Immuno-fluorescence (DNA fibers)
anti-ClDU/BrdU	BU1/75 (ICR1)	Abcam	ab6326	1/100	Immuno-fluorescence (DNA fibers)
Goat anti-Rat IgG (H+L) Cross-Adsorbed Secondary Antibody, Alexa Fluor™ 555	n/a	Invitrogen	A21434	1/200	Immuno-fluorescence (DNA fibers)
Goat anti-Mouse IgG (H+L) Cross-Adsorbed Secondary Antibody, Alexa Fluor™ 488	n/a	Invitrogen	A11001	1/100	Immuno-fluorescence (DNA fibers)
