# Supplementary material for: RAD51C-XRCC3 structure and cancer patient mutations define DNA replication roles
Source: Nat Commun. 2023 Jul 24;14:4445. doi: 10.1038/s41467-023-40096-1 (PMC10366140; doi:10.1038/s41467-023-40096-1)
Supplement: Supplementary file 3 — Reporting Summary [file 41467_2023_40096_MOESM3_ESM.pdf]

nature portfolio

Corresponding author(s): Katharina Schlacher

Last updated by author(s): 18.06.2023

Reporting Summary

Nature Portfolio wishes to improve the reproducibility of the work that we publish. This form provides structure for consistency and transparency in reporting. For further information on Nature Portfolio policies, see our Editorial Policies and the Editorial Policy Checklist.

Statistics

For all statistical analyses, confirm that the following items are present in the figure legend, table legend, main text, or Methods section.

n/a	Confirmed	
☐	☒	The exact sample size (*n*) for each experimental group/condition, given as a discrete number and unit of measurement
☒	☐	A statement on whether measurements were taken from distinct samples or whether the same sample was measured repeatedly
☐	☒	The statistical test(s) used AND whether they are one- or two-sided<br>*Only common tests should be described solely by name; describe more complex techniques in the Methods section.*
☒	☐	A description of all covariates tested
☒	☐	A description of any assumptions or corrections, such as tests of normality and adjustment for multiple comparisons
☐	☒	A full description of the statistical parameters including central tendency (e.g. means) or other basic estimates (e.g. regression coefficient) AND variation (e.g. standard deviation) or associated estimates of uncertainty (e.g. confidence intervals)
☐	☒	For null hypothesis testing, the test statistic (e.g. *F*, *t*, *r*) with confidence intervals, effect sizes, degrees of freedom and *P* value noted<br>*Give P values as exact values whenever suitable.*
☒	☐	For Bayesian analysis, information on the choice of priors and Markov chain Monte Carlo settings
☒	☐	For hierarchical and complex designs, identification of the appropriate level for tests and full reporting of outcomes
☒	☐	Estimates of effect sizes (e.g. Cohen's *d*, Pearson's *r*), indicating how they were calculated

Our web collection on statistics for biologists contains articles on many of the points above.

Software and code

Policy information about availability of computer code

Data collection

All X-ray crystallography data was collected at beamline 8.31 at the Advanced Light Source, Berkeley USA.
apCX3 X-ray crystallography data processing, structure solution, model building and refinement used XDS (Version January 26, 2018), PHASER ((1.20.1-4487) and COOT (0.9.6). apRAD51C NTD X-ray crystallography data processing, structure solution, model building and refinement used Moflm (7.0) and SCALA (3.3). SHELXD (-2012) Phenix (1.20.1-4487) and COOT (0.7.2). apRAD51C X-ray crystallography data processing, structure solution, model building and refinement used Moflm (7.0), SCALA (3.3), within the CCP4 suite (6.4.0) PHASER (2.4) and REFMAC (5.7), and COOT (0.7.2).

SAXS data was collected at Sibyls beamline 12.3.1. at the Advanced Light Source, Berkeley USA. SAXS data was processed with Scatter (v3) and Primus within the ATSAS suite (v3.2.1). hRAD51C-XRCC3 models were built with iTasser (v5.2) and molecular dynamics and minimal ensemble search done in BilboMD (v1). SAXS data is deposited within the small-angle scattering biological databank (SASDBD) and will be released upon publication.

Data analysis

Described in the manuscript if applicable

For manuscripts utilizing custom algorithms or software that are central to the research but not yet described in published literature, software must be made available to editors and reviewers. We strongly encourage code deposition in a community repository (e.g. GitHub). See the Nature Portfolio guidelines for submitting code & software for further information.

Data

Policy information about availability of data

All manuscripts must include a data availability statement. This statement should provide the following information, where applicable:

- Accession codes, unique identifiers, or web links for publicly available datasets
- A description of any restrictions on data availability
- For clinical datasets or third party data, please ensure that the statement adheres to our policy

All data pertaining to the results are available in the main text, the Supplementary material, and the source data file. The Genbank accession codes for Alvinella pompejana RAD51C and XRCC3 are OQ586110 and OQ586109, respectively, and will be publicly available prior to publication. The crystallographic models and data have been deposited in the protein databank with the following PDB accession codes: 8GJ9 (apRAD51C N-terminal domain), 8GJ8 (apRAD51C C-terminal domain), and 8GJA (apRAD51C-XRCC3 core). Coordinates will be publicly released upon publication.
Material requests underlie compliance with institutional policies and can be made by contacting the corresponding authors.

Research involving human participants, their data, or biological material

Policy information about studies with human participants or human data. See also policy information about sex, gender (identity/presentation), and sexual orientation and race, ethnicity and racism.

Reporting on sex and gender	*Use the terms sex (biological attribute) and gender (shaped by social and cultural circumstances) carefully in order to avoid confusing both terms. Indicate if findings apply to only one sex or gender; describe whether sex and gender were considered in study design; whether sex and/or gender was determined based on self-reporting or assigned and methods used.*<br>*Provide in the source data disaggregated sex and gender data, where this information has been collected, and if consent has been obtained for sharing of individual-level data; provide overall numbers in this Reporting Summary. Please state if this information has not been collected.*<br>*Report sex- and gender-based analyses where performed, justify reasons for lack of sex- and gender-based analysis.*
Reporting on race, ethnicity, or other socially relevant groupings	*Please specify the socially constructed or socially relevant categorization variable(s) used in your manuscript and explain why they were used. Please note that such variables should not be used as proxies for other socially constructed/relevant variables (for example, race or ethnicity should not be used as a proxy for socioeconomic status).*<br>*Provide clear definitions of the relevant terms used, how they were provided (by the participants/respondents, the researchers, or third parties), and the method(s) used to classify people into the different categories (e.g. self-report, census or administrative data, social media data, etc.)*<br>*Please provide details about how you controlled for confounding variables in your analyses.*
Population characteristics	*Describe the covariate-relevant population characteristics of the human research participants (e.g. age, genotypic information, past and current diagnosis and treatment categories). If you filled out the behavioural & social sciences study design questions and have nothing to add here, write "See above."*
Recruitment	*Describe how participants were recruited. Outline any potential self-selection bias or other biases that may be present and how these are likely to impact results.*
Ethics oversight	*Identify the organization(s) that approved the study protocol.*

Note that full information on the approval of the study protocol must also be provided in the manuscript.

Field-specific reporting

Please select the one below that is the best fit for your research. If you are not sure, read the appropriate sections before making your selection.

☒ Life sciences ☐ Behavioural & social sciences ☐ Ecological, evolutionary & environmental sciences

For a reference copy of the document with all sections, see nature.com/documents/nr-reporting-summary-flat.pdf

Life sciences study design

All studies must disclose on these points even when the disclosure is negative.

Sample size	Described in manuscript where appropriate, sample size was not predetermined, sample sizes were chosen as established by the field for given asssays.
Data exclusions	No quantifiable data was excluded, replications were successful
Replication	Sample sizes are described i the manuscript, experiments performed with 2-5 biological replicas except DNA binding by fluorescence polarization, which used used a n=3 technical replicate.
Randomization	Described in the manuscript where appropriate or not applicable.

Blinding	Described in the manuscript when applicable, samples were analyzed in a blinded fashion when possible and as established by the field for given assay

Reporting for specific materials, systems and methods

We require information from authors about some types of materials, experimental systems and methods used in many studies. Here, indicate whether each material, system or method listed is relevant to your study. If you are not sure if a list item applies to your research, read the appropriate section before selecting a response.

Materials & experimental systems

n/a	Involved in the study	
☐	☒	Antibodies
☐	☒	Eukaryotic cell lines
☒	☐	Palaeontology and archaeology
☐	☒	Animals and other organisms
☒	☐	Clinical data
☒	☐	Dual use research of concern
☒	☐	Plants

Methods

n/a	Involved in the study	
☒	☐	ChIP-seq
☒	☐	Flow cytometry
☒	☐	MRI-based neuroimaging

Antibodies

Antibodies used	Detailed in manuscript (Supplementary table 3
Validation	the validation is performed as part of the study and shown in the manuscript

Eukaryotic cell lines

Policy information about cell lines and Sex and Gender in Research

Cell line source(s)	HAP1 cells (cat# C631, Horizon Genomics GmbH)
Authentication	Sanger sequencing and STR profiling.
Mycoplasma contamination	Cells used in this study tested negative for mycoplasma.
Commonly misidentified lines (See ICLAC register)	No commonly misidentified cell lines were used in this study.

Animals and other research organisms

Policy information about studies involving animals; ARRIVE guidelines recommended for reporting animal research, and Sex and Gender in Research

Laboratory animals	NOD.Cg-Prkdc"id Jl2rg,mlWjl/SzJ, 8-12 week old were used.
Wild animals	No wild animals were used.
Reporting on sex	these are xenograft studies, gender of the host is not applicable and generally not considered in the field for xenograft studies
Field-collected samples	No field collected samples were used in the study.
Ethics oversight	The animal experiments were approved by the institutional animal care and use committee (IACUC) and was described in an Animal Care and Use Form (ACUF, protocol nr 00001436-RN01). All procedures and methods were performed according to the federal and state regulations as well as MD Anderson Cancer Center institutional guidelines and policies for the protection of animals.

Note that full information on the approval of the study protocol must also be provided in the manuscript.
